# A SARS-CoV-2 related signature that explores the tumor microenvironment and predicts immunotherapy response in esophageal squamous cell cancer

**DOI:** 10.18632/aging.205090

**Published:** 2023-10-06

**Authors:** Qianhe Ren, Pengpeng Zhang, Shengyi Zhang, Wenhui Chen, Hao Chi, Wei Wang, Wei Zhang, Haoran Lin, Yue Yu

**Affiliations:** 1 Department of Thoracic Surgery, The First Affiliated Hospital of Nanjing Medical University, Nanjing, China; 2 Department of Radiology, The First Affiliated Hospital of Nanjing Medical University, Nanjing, China; 3 Department of Thoracic Surgery, Songjiang Hospital Affiliated to Shanghai Jiaotong University School of Medicine (Preparatory Stage), Shanghai, China; 4 Department of Otolaryngology Head and Neck Surgery, Beijing TongRen Hospital, Capital Medical University, Beijing, China; 5 School of Clinical Medical Sciences, Southwest Medical University, Luzhou, China

**Keywords:** esophageal squamous cell carcinoma, SARS-CoV-2, risk signature, prognosis, immunotherapy

## Abstract

Background: The existing therapeutic approaches for combating tumors are insufficient in completely eradicating malignancy, as cancer facilitates tumor relapse and develops resistance to treatment interventions. The potential mechanistic connection between SARS-CoV-2 and ESCC has received limited attention. Therefore, our objective was to investigate the characteristics of SARS-CoV-2-related-genes (SCRGs) in esophageal squamous cancer (ESCC).

Methods: Raw data were obtained from the TCGA and GEO databases. Clustering of SCRGs from the scRNA-seq data was conducted using the Seurat R package. A risk signature was then generated using Lasso regression, incorporating prognostic genes related to SCRGs. Subsequently, a nomogram model was developed based on the clinicopathological characteristics and the risk signature.

Results: Eight clusters of SCRGs were identified in ESCC utilizing scRNA-seq data, of which three exhibited prognostic implications. A risk signature was then made up with bulk RNA-seq, which displayed substantial correlations with immune infiltration. The novel signature was verified to have excellent prognostic efficacy.

Conclusion: The utilization of risk signatures based on SCRGs can efficiently forecast the prognosis of ESCC. A thorough characterization of the SCRGs signature in ESCC could facilitate the interpretation of ESCC's response to immunotherapy and offer innovative approaches to cancer therapy.

## INTRODUCTION

Esophageal carcinoma is a widespread malignancy on a global scale, and it is ranked as the sixth foremost cause of cancer-related mortality [1]. Esophageal squamous cell carcinoma (ESCC) is the primary histological subtype that prevails in cases of esophageal cancer in China, constituting over 90% of occurrences [2]. Despite the progress made in diagnostic and therapeutic approaches, this malignancy still demonstrates a discouraging prognosis, with a 5-year survival rate ranging between 15% and 25% [3]. The high mortality rate linked to esophageal carcinoma can be attributed to the insufficiency of effective diagnostic and therapeutic modalities [4, 5]. The elevated mortality rates can also be attributed to chemotherapy resistance. Numerous investigations have showcased that apoptosis disruption, aberrant autophagy, heightened DNA repair mechanisms, epithelial–mesenchymal transition facilitation, deactivation of drug metabolism enzymes, and alterations in the expression or functionality of membrane transporters are intricately linked to the evolution of drug resistance [6]. Therefore, it is crucial to augment our understanding of the molecular mechanisms underlying the progression of esophageal malignancies in order to facilitate the clinical diagnosis and management of affected individuals. In 2020, a global pandemic was unleashed by SARS-CoV-2, the causative agent of COVID-19. SARS-CoV-2 belongs to the genus Betacoronavirus and is characterized as an enveloped, single-stranded RNA virus with a positive-sense genome [7]. The roles carried out by SARS-CoV-2 proteins (such as ADRP, PLpro, DMV, among others) encompass safeguarding this pathogen, aiding in its attachment to host cells, obstructing the expression of host genes, and eluding the innate immune response—thereby facilitating the virus’s replication and propagation. Over the preceding year, structural biologists have unveiled the pivotal configurations of both individual proteins and their complexes within SARS-CoV-2. This revelation offers an atomic-scale comprehension of the diverse processes unfolding in the viral life cycle. These structural insights hold significance not solely for deciphering the intricate mechanisms through which proteins and their assemblies execute distinct toggling or regulatory roles, but also for shedding light on the strategies employed by the virus to evade the immune system [8]. Coronaviruses exhibit a propensity for substantial genetic recombination and mutation rates, thereby leading to a wide range of ecological diversification [9]. In addition to affecting the respiratory and central nervous systems, SARS-CoV-2 also has significant effects on the digestive system, particularly the esophagus [10–12]. The infection induced by SARS-CoV-2 requires the presence of crucial receptors, namely Angiotensin I-converting enzyme 2 (ACE2), along with type II transmembrane serine protease 2 and 4 (TMPRSS2 and TMPRSS4). These receptors are expressed in different tissues or organs of tree shrews, with the kidney displaying the most elevated ACE2 expression, followed by the lung and liver. Conversely, relatively high expression of TMPRSS2 and TMPRSS4 is observed in the esophagus, lung, liver, intestine, and kidney [13]. Our hypothesis suggests that the expression levels of genes associated with SARS-CoV-2 (SCRGs) may undergo a significant increase in the esophagus. Contemporary literature has allocated limited attention to examining the connection between SARS-CoV-2 and the potential for cancer development. Employing Mendelian randomization (MR), we undertook an investigation into the underlying causal relationships between the three distinct forms of SARS-CoV-2 exposure (namely, critically ill COVID-19, hospitalized COVID-19, and infection by the respiratory syndrome coronavirus 2, SARS-CoV-2) and 33 varied categories of cancers prevalent within the European population. The outcomes derived from the inverse-variance-weighted model have illuminated that the genetic predisposition towards critically ill COVID-19 exhibits suggestive and causal links to an elevated susceptibility to several forms of malignancies. Notable among these are esophageal cancer, HER2-positive breast cancer, colorectal cancer, gastric cancer, and colon cancer. Under the circumstances, more efficient diagnostic and therapeutic methods could be provided for esophageal cancer [14]. However, there is a lack of comprehensive investigations examining the correlation between SCRGs and the progression of esophageal cancer. In recent decades, the research on the development of esophageal squamous cell carcinoma (ESCC) has predominantly centered around the mutations and malignant transformation of squamous cells within the esophageal epithelium. Intriguingly, through extensive genomic analysis, certain mutated genes implicated in the regulation of the cell cycle and apoptosis, such as CCND1, CDKN2A, SOX2, and TP53, have been identified in a subgroup of ESCC patients [15]. Despite the thorough genomic characterization of individuals diagnosed with esophageal squamous cell carcinoma (ESCC), the translation of these discoveries into clinical application remains inadequate, offering limited advantages to patients. The existence of genomic and epigenomic heterogeneity, both intra- and inter-tumor, may partially contribute to this constraint [16]. Conversely, a growing body of evidence indicates that the interplay between mutated cells and immune cells within the tissue microenvironment exerts a direct influence on, and potentially governs, the advancement of cancer management [17]. Considering the systemic toxicity and multidrug resistance patients have during traditional chemotherapy, immunotherapy functions as a promising modality for cancer treatment, having anti-tumor effects and increasing the OS of patients with various cancers [18]. Notably, immunotherapies directed at the tumor microenvironment, as opposed to the intrinsic tumor cells, have exhibited remarkable efficacy in combating diverse cancer types, thereby presenting promising novel treatment modalities for esophageal squamous cell carcinoma (ESCC) [19]. The approval by the US Food and Drug Administration (FDA) of interferon-alpha2 anti-tumor cytokine marked the initiation of these advancements [20]. Since then, the scope of immunotherapeutic drugs has expanded to include anti-tumor cytokines, checkpoint inhibitors, adoptive transfer T-cell therapy, and cancer vaccines [21]. Furthermore, a growing array of immunotherapeutic agents has undergone rigorous clinical trials and been incorporated into the domain of clinical implementation. The fundamental principle of immunotherapy acknowledges the interconnectedness between tumors and their surrounding tumor microenvironment, which includes host immune cells. It posits the ability to harness the immune system to initiate anti-tumor responses [22].

In this study, single-cell RNA sequencing (scRNA-seq) data and transcriptome data retrieved from publicly available databases were employed to discern subclusters of SARS-CoV-2-related genes (SCRGs) and establish an ESCC risk signature based on these genes. The clinical significance of the SCRGs-based signature was evaluated, alongside an analysis of the immune landscape and an assessment of its immunotherapeutic potential. Furthermore, a novel nomogram was developed by integrating the risk signature with clinicopathological characteristics, thereby facilitating the clinical utilization of SCRGs in prognosticating ESCC. Our findings have the potential to provide novel insights into the pathophysiology of ESCC, enabling more personalized therapeutic strategies and improved outcomes for individuals affected by this condition.

## METHODS

### Data collection and processing

The ESCC single-cell RNA sequencing (scRNA-seq) data was obtained from the Gene Expression Omnibus (GEO) database (accession number GSE196756). Two cohorts, namely GSE53624 and TCGA data, were chosen for subsequent analysis. Cells with fewer than 250 expressed genes or genes expressed in less than three cells were excluded. The PercentageFeatureSet function from the Seurat R package was utilized to determine the percentage of rRNA and mitochondria, resulting in a final count of 12,118 cells for further analysis.

Additionally, transcriptomic data and corresponding clinical data of esophageal squamous cell carcinoma (ESCC) were retrieved from The Cancer Genome Atlas (TCGA) database. Samples lacking outcome status or survival data were excluded, resulting in 94 ESCC samples for external validation. The training cohort consisted of 119 tumor samples and 119 normal samples from the GSE53624 dataset obtained from the Gene Expression Omnibus (GEO) database, after excluding samples without follow-up information. Based on relevant literature, the gene expression profiles of ten cancer-associated pathways (Cell Cycle, NRF1, MYC, NOTCH, HIPPO, PI3K, TP53, PI3K, WNT, and TGF-Beta) were analyzed in our dataset.

### SCRGs identification

The Seurat package was employed to conduct a comprehensive analysis of esophageal squamous cell carcinoma (ESCC) single-cell RNA sequencing (scRNA-seq) data, aiming to evaluate the gene expression profiles of scRNA-seq genes (SCRGs) associated with ESCC. The initial data preprocessing involved excluding cells with gene expression below 250 or above 6000, followed by log-normalization of the remaining expressed genes. To address potential batch effects from the four samples, the FindIntegrationAnchors function was utilized. Dimensionality reduction was achieved using a non-linear t-distributed Stochastic Neighbor Embedding (tSNE) method with a resolution of 0.1 and selection of 30 principal components. The categorization of individual cells into distinct subgroups was performed using the FindNeighbors and FindClusters functions with a dimensional parameter of 30 and a resolution of 0.1. Further tSNE dimensional reduction was carried out using the RuntSNE function. The SCRGs were annotated using NLRX1 and SLC9A3R1 as marker genes, followed by re-clustering using the FindClusters and FindNeighbors functions. Marker genes for each SCRG cluster were identified using the FindAllMarkers function, comparing different clusters based on parameters such as minpct, logFC, and adjust *p*-value. Finally, the CopyKAT R package was utilized to analyze the copy number variation (CNV) characteristics of the SCRG clusters, enabling the differentiation between tumor cells and normal cells.

### Hub genes identification according to SCRGs

The limma package was utilized to identify differentially expressed genes (DEGs) between normal and tumor tissues, applying criteria of |log2(FoldChange)|>1 and FDR <0.05. Subsequently, the correlations between SCRGs clusters and DEGs were evaluated to identify pivotal SCRGs with *p* < 0.01 and cor >0.4. Prognosis-related genes were determined through univariate Cox regression analysis using the survival package, followed by lasso regression (with lambda = 0.0595) for gene count reduction. The SCRG-based risk signature was established through multivariate Cox regression analysis employing the stepwise regression method. The risk signature was calculated using a formula involving multiple genes and normalized to stratify patients into low- and high-risk groups. The prognostic value of the risk signature was assessed using the timeROC package for ROC analysis, revealing its significant prognostic relevance for patients. Our analysis yields valuable insights into the molecular mechanisms underlying tumor development and underscores the potential of SCRGs as prognostic biomarkers for cancer patients.

### Developing a novel nomogram founded on risk signature

After conducting univariate and multivariate Cox regression analyses incorporating the risk signature and clinicopathological characteristics, an innovative nomogram was constructed to prognosticate the outcome of ESCC. This was achieved by selecting variables with *p*-values below 0.05 in the multivariate Cox model. A calibration curve was generated to evaluate the predictive precision of the model.

### Immune landscape in ESCC

The association between the tumor immune microenvironment (TIME) and the risk signature was thoroughly examined using multiple algorithms, including CIBERSORT, EPIC, MCPCOUNTER, and TIMER. Various metrics were employed to quantify the heterogeneity of the tumor microenvironment, such as stromal scores, immune scores, and estimate scores (obtained by summing stromal scores and immune scores), utilizing the “estimate” R package. Additionally, the CIBERSORT algorithm was utilized to estimate the proportions of 22 immune cell subtypes within the GSE53624 cohort. Furthermore, the correlation between the signature genes and immune scores was investigated to illuminate the significant influence of these genes on immune-related functions.

### Response to immunotherapy

Subsequently, transcriptomic data and relevant clinical data were acquired from patients enrolled in the IMvigor210 cohort, who were treated with anti-PD-L1 therapy. This was done to evaluate the predictive capacity of our risk signature in determining the responsiveness to immunotherapy, specifically immune checkpoint blockade. Additionally, transcriptomic data from the GSE78220 cohort, consisting of melanoma patients who underwent anti-PD-1 checkpoint inhibition therapy, were obtained for further analysis.

### Consensus clustering and drug sensitivity analysis

The heterogeneity of esophageal squamous cell carcinoma (ESCC) was examined by categorizing patients into distinct clusters based on the expression of single-cell RNA sequencing genes (SCRGs), utilizing the ‘ConsensusClusterPlus’ R package. Survival, tumor immune microenvironment (TIME), and immune checkpoints were compared between the subgroups. An immune landscape heatmap was generated to visualize the variations among ESCC patients in different clusters. To evaluate the clinical relevance of the risk model in ESCC treatment, the IC50 values of commonly used chemotherapeutic agents in the GSE53624 dataset were calculated using the ‘pRRophetic’ R package. Violin plots were used to compare the IC50 values of different antitumor drugs between the cluster groups. These statistical models allow the prediction of clinical chemotherapeutic response solely based on baseline tumor gene expression data and drug sensitivity data derived from cell lines in the Cancer Genome Project.

### RNA isolation and quantitative RT-PCR (qRT-PCR) assay

Total RNA from esophageal squamous cell carcinoma (ESCC) cells or tissues was extracted using TRIzol reagent (Thermo Fisher Scientific, Waltham, MA, USA). Complementary DNA (cDNA) synthesis was conducted according to the manufacturer’s instructions, employing the RevertAid™ First Strand cDNA Synthesis Kit (Thermo Fisher Scientific). Subsequently, quantitative reverse transcription PCR (qRT-PCR) was carried out using a SYBR Green PCR kit (Takara Bio, Otsu, Japan) on a StepOne Real-Time PCR system (Thermo Fisher Scientific). The 2−ΔΔCT method was utilized to quantify the relative gene expression levels.

### Statistical analysis

All statistical analyses were performed using R software (version 4.1.0). The Wilcoxon test was employed to compare groups, while correlation matrices were evaluated using either Spearman or Pearson correlation. Survival differences were assessed using the Log-rank test and visualized with Kaplan-Meier curves, considering a *p*-value < 0.05 as statistically significant.

### Data availability statement

The data that support the findings of this study are available from the corresponding author upon reasonable request. We have uploaded all the raw data, code and images to the Jianguo Yun. This data is easily access at the following link: https://www.jianguoyun.com/p/DbCR0TIQjdemCxiu4IIFIAA.

## RESULTS

### Screening the SCRGs based on scRNA-seq samples

**Figure 1:**
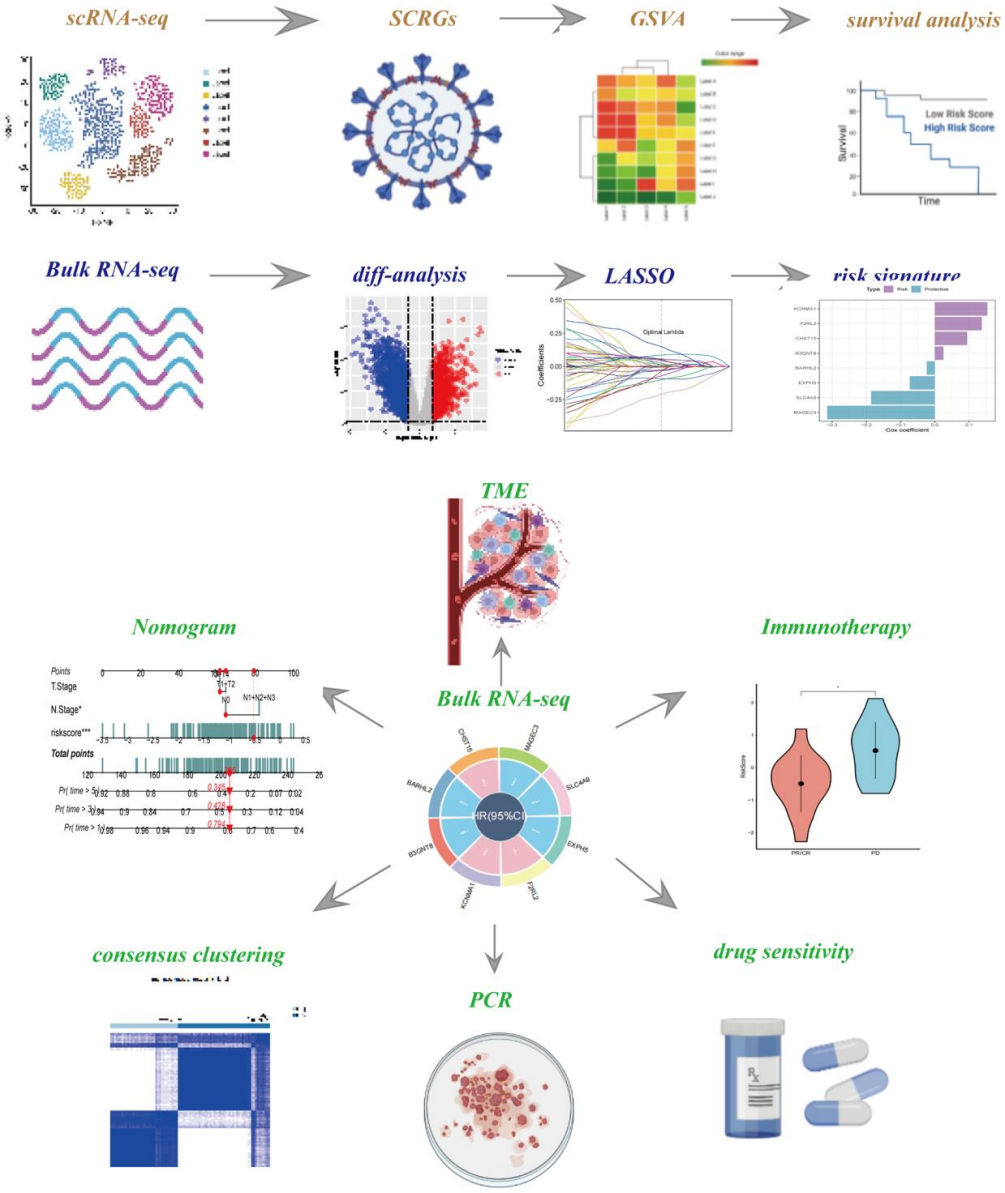
The flow chart of this study.

**Figure 2:**
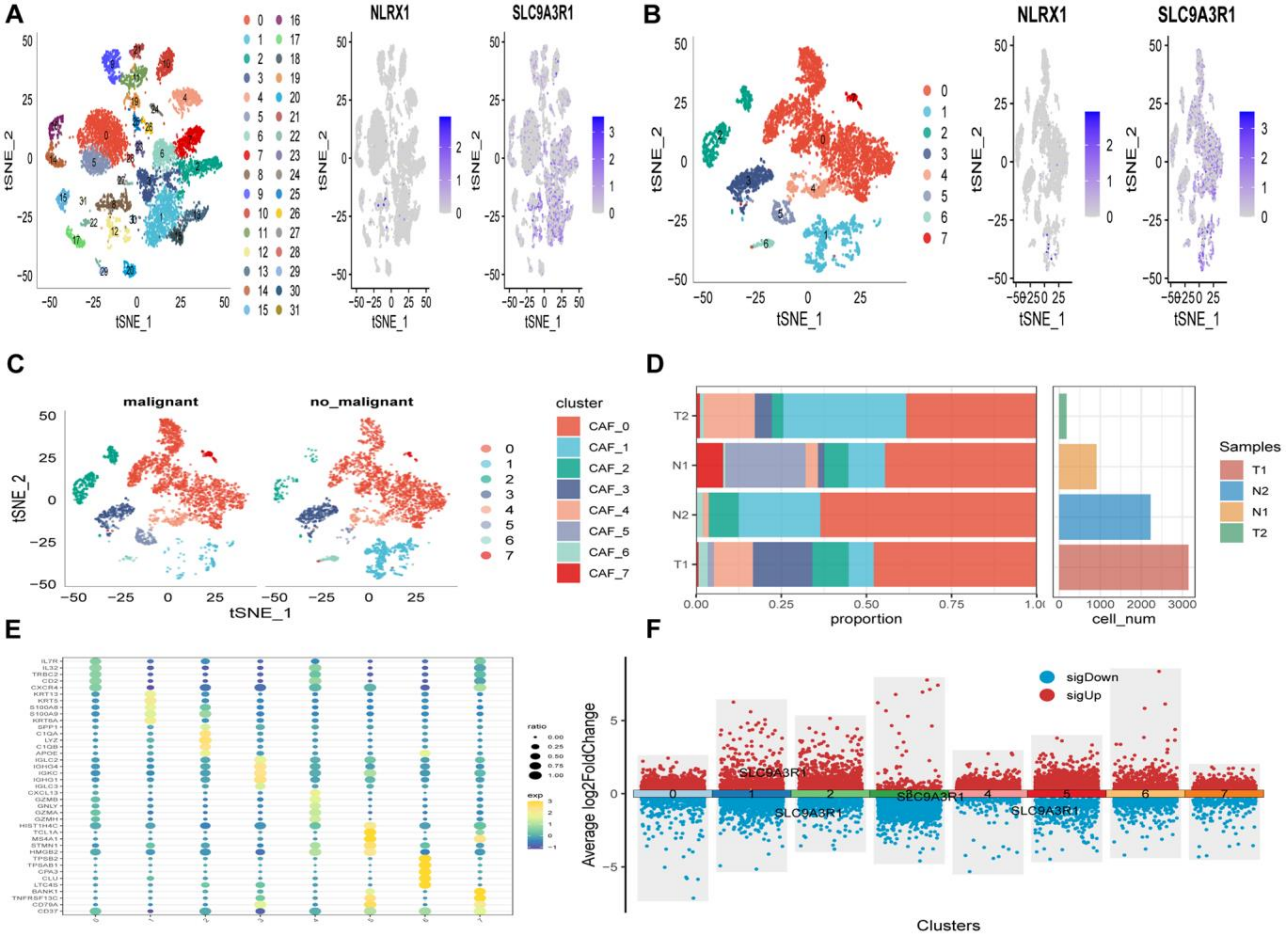
**The identification of SCRGs clusters according to scRNA data of ESCC patients.** (**A**) tSNE plots of distribution of 32 clusters and SARS-CoV-2 marker genes expression. (**B**) tSNE plots of distributions of eight fibroblasts after clustering. (**C**) tSNE distribution of malignant and non-malignant cells predicted by copycat package. (**D**) Subgroups in cancer and adjacent tissue and proportion as well as cell number calculation. (**E**) Bubble diagram of the top5 marker gene expression of subgroups. (**F**) Volcano plot of the top5 marker gene expression of subgroups.

The study’s flowchart is presented in Figure 1. An analysis of scRNA-seq data yielded a total of 18,024 cells. Further details on data preprocessing can be found in Supplementary Figure 1. After log-normalization and dimensionality reduction, 32 distinct subpopulations were identified, as depicted in Figure 2A. Additionally, based on the expression of NLRX1 and SLC9A3R1 as marker genes, eight distinct SCRG populations were delineated and visualized in Figure 2B. The distributional differences between tumor and normal cells within these eight SCRG clusters are illustrated in Figure 2C. The relative proportions of these eight clusters in each cohort were calculated and presented in histograms shown in Figure 2D. Moreover, the expression levels of the top five DEGs were visualized using bubble diagrams (Figure 2E) and volcano plots (Figure 2F).

### The cancer-related pathways in SCRGs

**Figure 3:**
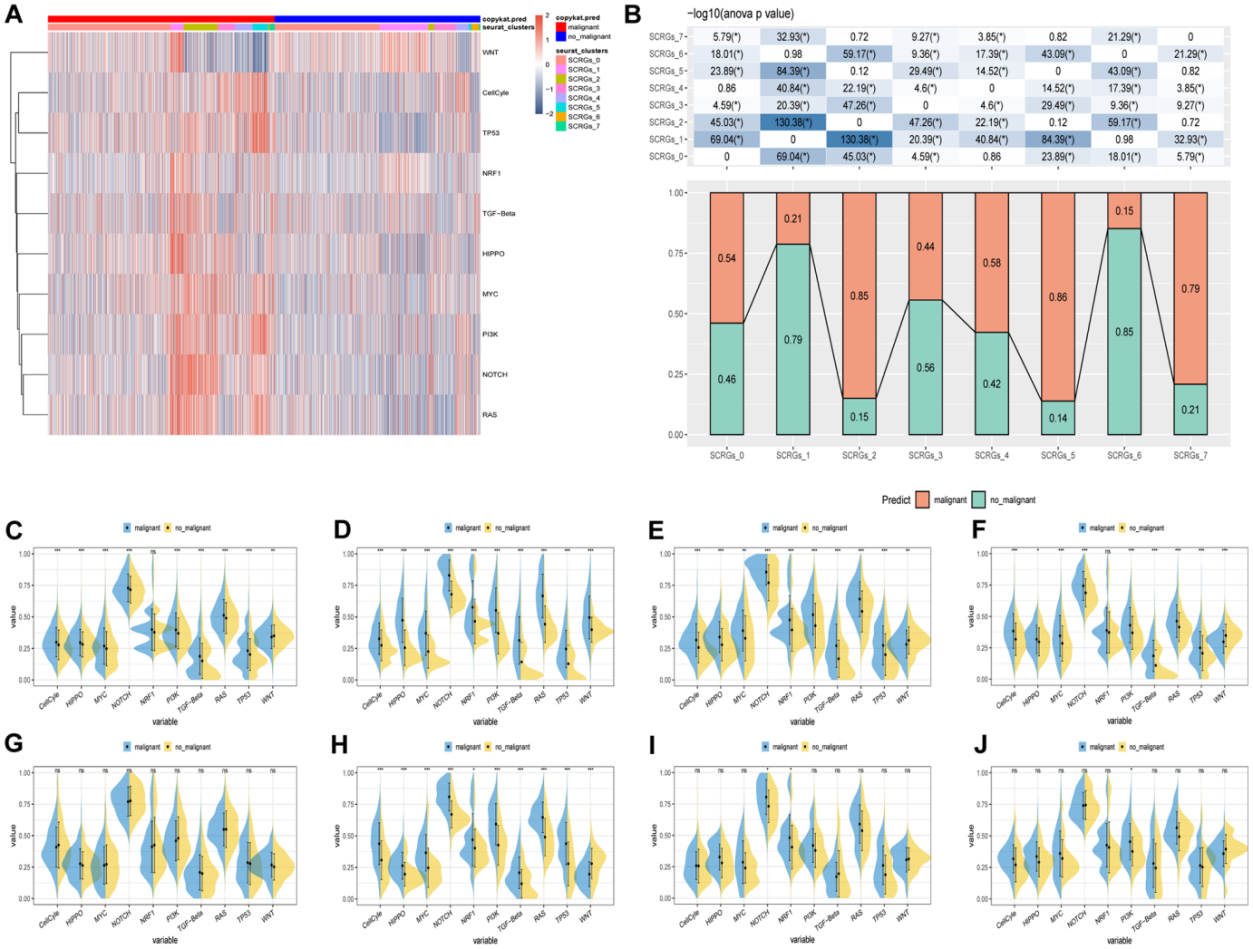
**The characteristics of tumor-associated pathways in SCRGs clusters.** (**A**) Heatmap of 10 tumor-associated pathways enriched in SCRGs cells. (**B**) Comparison between each cluster based on proportions of malignant and non-malignant cells. (**C**–**J**) Comparison of each pathway between malignant and non-malignant cells based on GSVA score in SCRGs clusters. (Wilcox. Test, ^*^P < 0.05; ^**^P < 0.01; ^***^P < 0.001; Abbreviation: ns: not significant).

The relationship between tumor progression and SCRGs clusters was investigated by examining the characteristics of ten pathways associated with tumor development in eight distinct clusters. Gene Set Variation Analysis (GSVA) scores were utilized to assess the variations in these pathways among the SCRGs clusters, as shown in Figure 3A. Notably, the SCRGs_3 cluster exhibited a significant difference in the proportion of malignant cells compared to the other clusters. Moreover, the SCRGs_2, SCRGs_5, and SCRGs_7 clusters displayed considerably higher proportions of malignant cells than the other clusters. In contrast, the SCRGs_1 and SCRGs_6 clusters demonstrated the highest proportion of non-malignant cells (Figure 3B). Additionally, a comparison of GSVA scores for the ten tumor-associated pathways was conducted to assess differences between malignant and non-malignant cells within each SCRGs cluster, revealing only minor disparities (Figure 3C–3J).

### Associations between SCRGs clusters and prognosis

**Figure 4:**
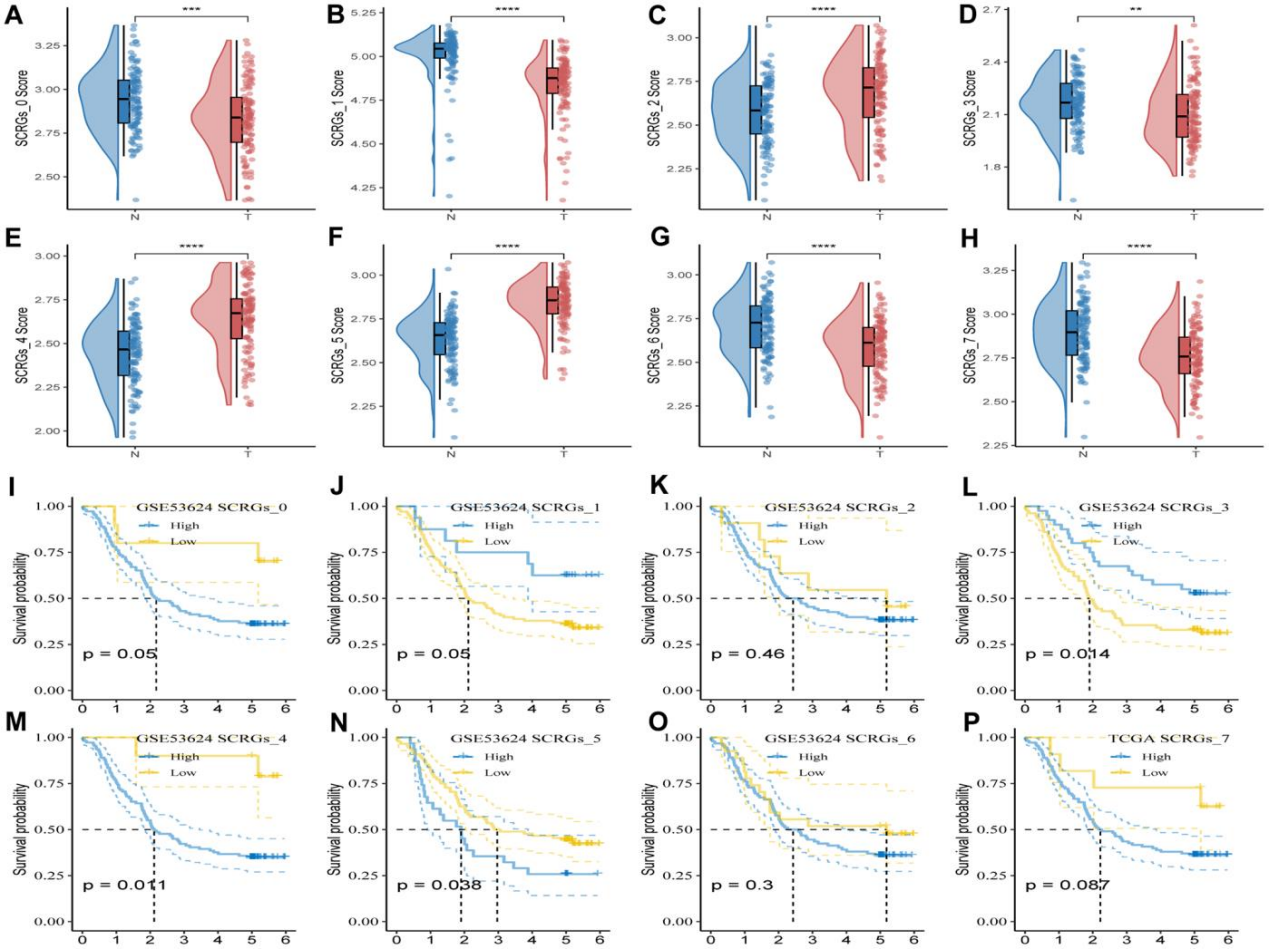
**GSVA analysis based on SCRGs clusters.** (**A**–**H**) Comparison of ssGSVA score based on each cluster between normal samples and tumor ones. (**I**–**P**) K-M curves of the high and low SCRGs score groups in the SCRGs clusters. (^**^P < 0.01; ^***^P < 0.001; ^****^P < 0.0001).

To evaluate the association between SCRGs clusters and prognosis, the ssGSEA score of the top five DEGs in each SCRGs cluster was calculated for individual samples in the GSE53624 cohort. The results revealed that tumor samples in SCRGs_2, SCRGs_4, and SCRGs_5 clusters exhibited significantly higher scores compared to normal samples. Conversely, the remaining SCRGs clusters demonstrated the opposite trend (Figure 4A–4H). Subsequently, utilizing the optimal cut-off value, the survminer R package was utilized to categorize ESCC samples from the GSE53624 dataset into high and low SCRGs score groups. Notably, significant differences were observed between the high- and low-SCRGs score groups in SCRGs_3, SCRGs_4, and SCRGs_5 clusters, while no correlation with ESCC prognosis was observed in other clusters (Figure 4I–4P). These findings suggest a potential significant role of SCRGs_3, SCRGs_4, and SCRGs_5 in the progression of ESCC.

### Hub genes identification correlated with SCRGs

**Figure 5:**
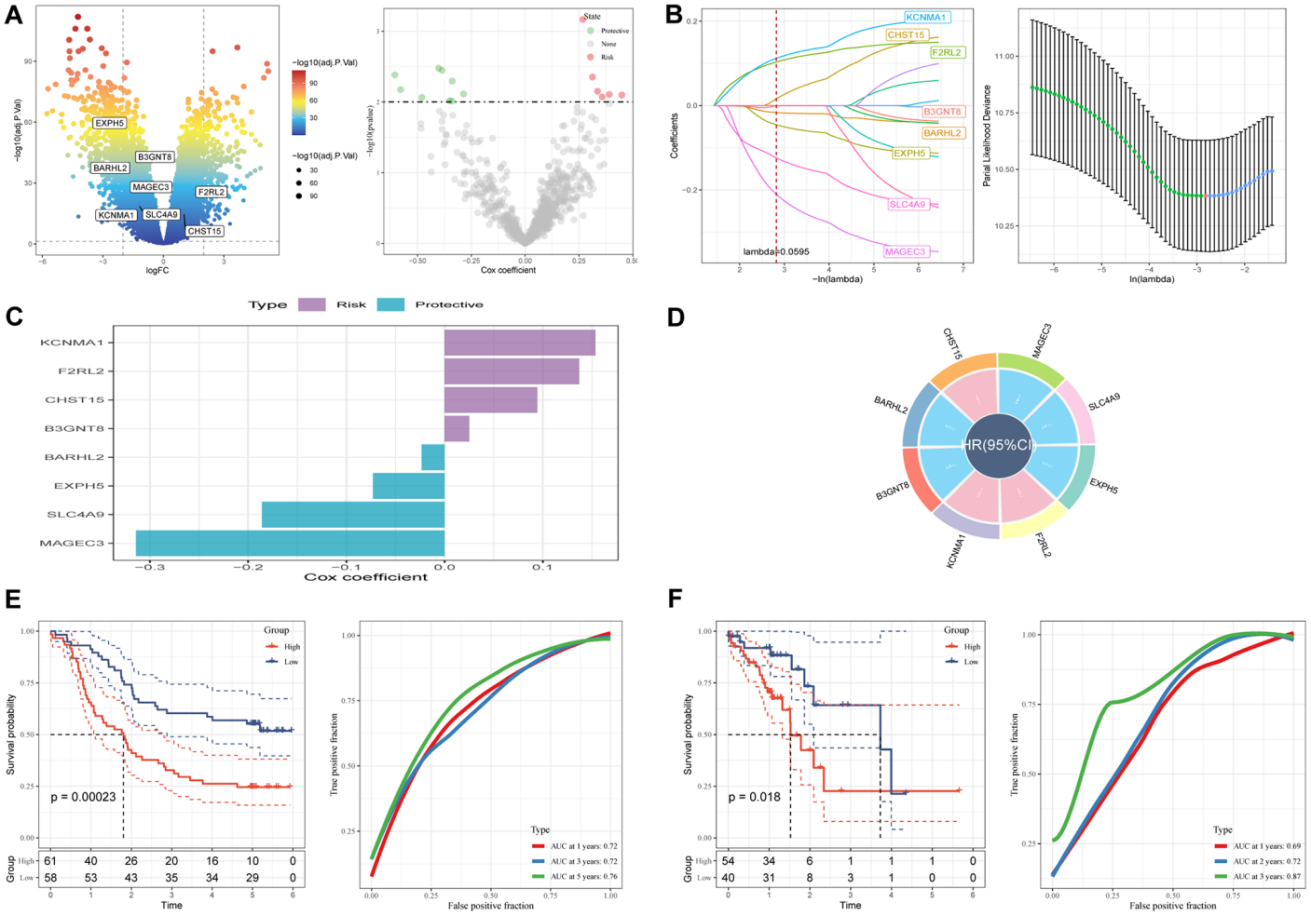
**A novel risk signature constructed based on several SCRGs.** (**A**) Volcano plot of differentially expressed genes between tumor and normal samples in GSE53624 cohort. Volcano plot of prognosis-correlated genes obtained by univariate Cox regression analysis. (**B**) Each independent variable’s trajectory and distributions for the lambda. (**C**) The multivariate Cox coefficients for each gene in the risk signature. (**D**) Circle plot showing each gene in the risk signature. (**E**) K-M and ROC curves of the risk signature in GSE53624 cohort. (**F**) K-M and ROC curves of the risk signature in TCGA cohort.

A prognostic signature was established by comparing tumor and normal samples, resulting in the identification of 17,080 DEGs, including 7,556 down-regulated and 9,524 up-regulated genes (Figure 5A). Out of these, 770 genes showed significant associations with prognosis-related SCRGs clusters. Univariate Cox regression analysis was conducted to evaluate the prognostic value of each gene, leading to the identification of 9 genes associated with protective factors and 7 genes correlated with risk values. Subsequently, Lasso Cox regression analysis was performed to reduce the number of genes (Figure 5B). Using the stepwise regression method, a risk signature comprising eight genes was constructed (Figure 5C, 5D): KCNMA1, F2RL2, CHST15, B3GNT8, BARHL2, EXPH5, SLC4A9, and MAGEC3. The risk signature was formulated as follows: “−0.073EXPH5 + 0.137F2RL2 + 0.154KCNMA1 + 0.025B3GNT8 + −0.024BARHL2 + 0.094CHST15 + −0.314MAGEC3 + −0.186SLC4A9”. The risk score for each sample was calculated using z-mean normalization, and patients were categorized into high and low-risk groups. Kaplan-Meier survival analysis demonstrated that patients in the high-risk clusters had poorer prognosis compared to those in the low-risk clusters in both the GSE53624 (Figure 5E) and TCGA cohorts (Figure 5F). Moreover, the model exhibited excellent predictive capability, as indicated by commendable AUC values in both cohorts. The distribution of patient survival outcomes, risk scores, and the expression of hub genes were illustrated in Supplementary Figure 2 for both GEO and TCGA cohorts, emphasizing the significantly unfavorable status of patients in the high-risk group. (A brief description of the sample size and characteristics used in the study was elucidated in file ‘Table 1’).

**Table 1:** Characteristics of different cohorts of ESCC patients.

**Variables**	**Group**	**Training set (*n* = 238)**	**Testing set (*n* = 95)**
**Age**	**≤65**	176	–
	**>65**	62	–
**Gender**	**Female**	42	15
	**Male**	196	80
**Vital status**	**Alive**	92	64
	**Dead**	146	31
**Survival time**		1111	459
**Clinical Stage**	**I**	12	7
	**II**	94	56
	**III**	132	26
	**IV**	–	4
	**Unknow**	–	2
**T stage**	**T1**	16	8
	**T2**	40	32
	**T3**	124	49
	**T4**	58	4
	**Tx**	–	2
**N stage**	**N0**	108	55
	**N1**	84	28
	**N2**	26	6
	**N3**	20	3
	**Nx**	–	3
**M stage**	**M0**	–	83
	**M1**	–	4
	**Mx**	–	8

### Recognition of independent risk factors and nomogram development

**Figure 6:**
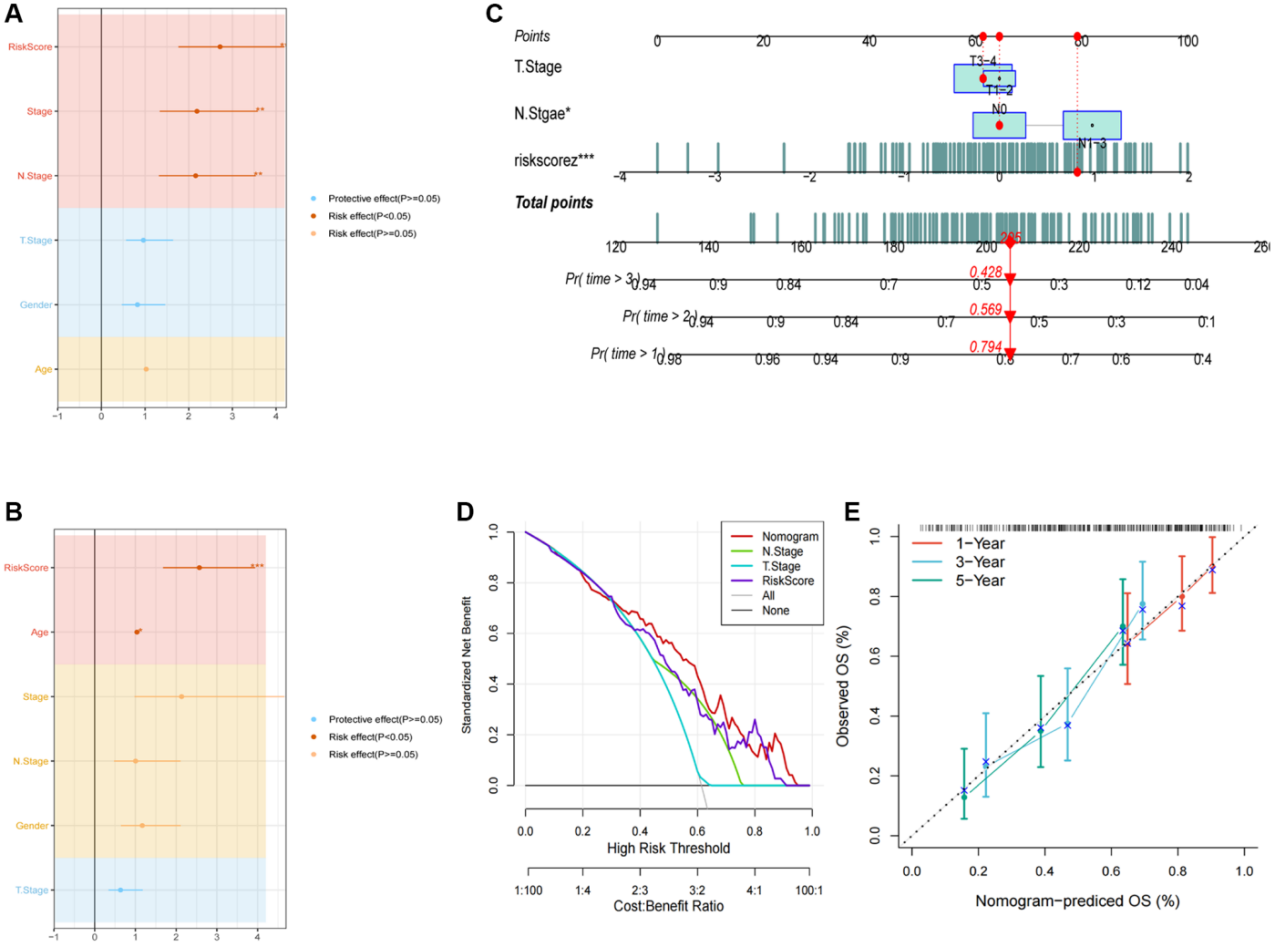
**Development of a novel nomogram integrating the risk signature and several clinicopathologic features.** (**A**) Results of univariate Cox regression analysis based on risk score and clinicopathologic features. (**B**) Results of multivariate Cox regression analysis based on risk score and clinicopathologic features. (**C**) Construction of the nomogram integrating the T, N-stage and risk score. (**D**) Decision curve for nomogram. (**E**) Calibration curves for 1, 2, and 3 years of nomogram. (^*^P < 0.05; ^**^P < 0.01; ^***^P < 0.001).

The integration of clinicopathological characteristics and risk score through univariate and multivariate Cox regression analyses has enhanced the precision of the prognostic model. The risk signature was identified as a significant independent prognosticator for ESCC, with a *p*-value below 0.001, as illustrated in Figure 6A, 6B. Furthermore, a novel nomogram incorporating T-stage, N-stage, and the risk score has been formulated, as depicted in Figure 6C. Through calibration and decision plot analysis, this nomogram exhibits robust predictive potential for actual survival outcomes, as indicated in Figure 6D, 6E.

### Pathway enrichment analysis

Gene Set Enrichment Analysis was conducted utilizing the eight genes encompassed in the risk signature, revealing a notable association with nine pathways (refer to Supplementary Figure 3A, 3B). R2RL2, KCNMA1, and CHST15 exhibited positive correlations with most pathways, except for neuroactive ligand receptor interaction and olfactory transduction, which have been implicated in suppressing the migration and progression of ESCC. The up-regulated genes predominantly enriched systemic lupus erythematosus and cytokine-cytokine receptor interaction, as depicted in Supplementary Figure 3C. Conversely, the down-regulated genes demonstrated significant correlations with linoleic acid metabolism and olfactory transduction. The findings of the GO analysis are presented in Supplementary Figure 3D–3F.

### Immune infiltrations landscape and correlation between risk genes and immunity

**Figure 7:**
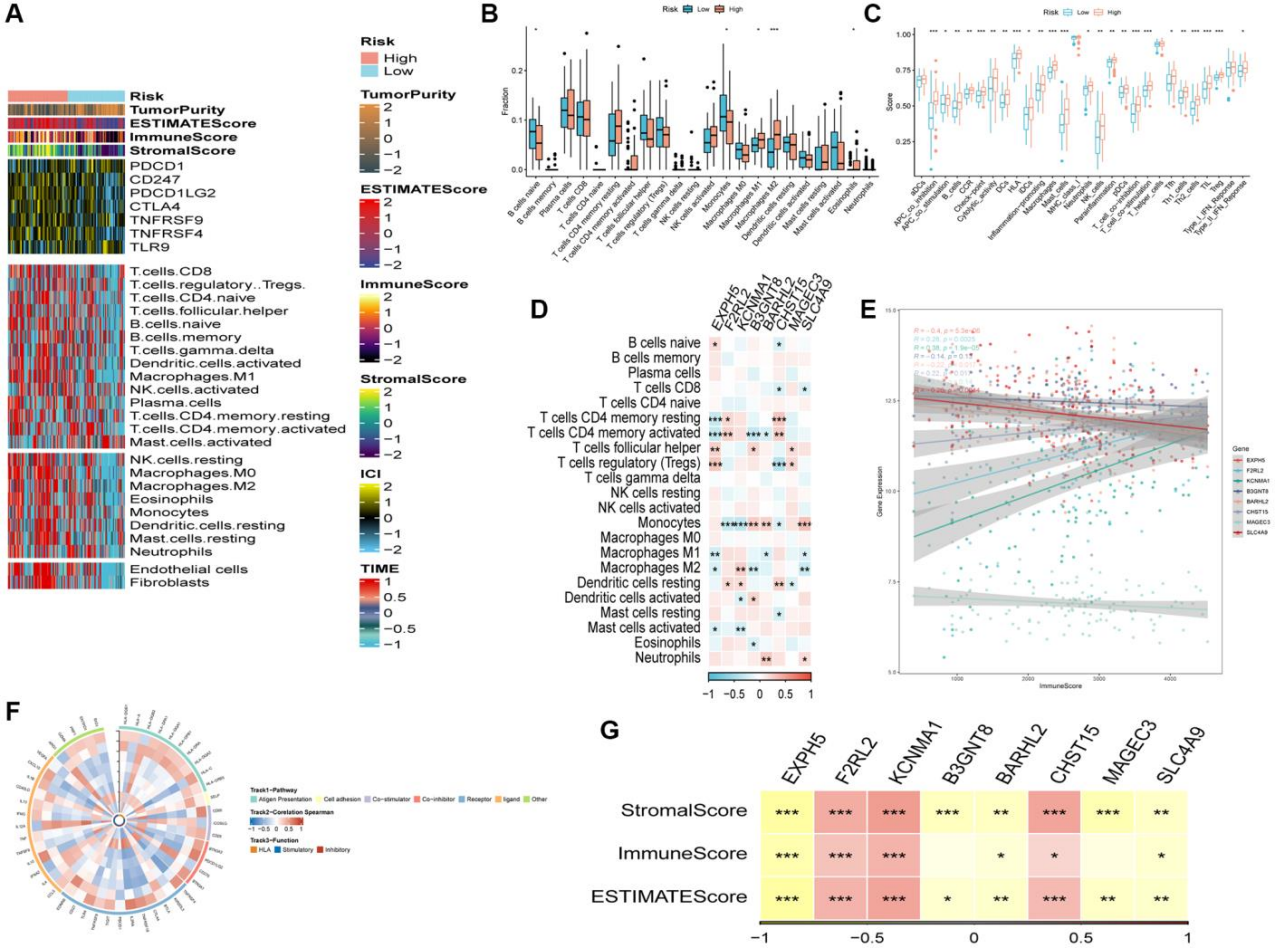
**The immune infiltrations analysis.** (**A**) Heatmap of results on immune cells of tumor microenvironment (TME) in ESCC with multialgorithm, including existing data from platform TIMER and MCP-counter. TME-related scores were exhibited in the top bar. (**B**) Comparison of proportions of 22 immune-related cells between high-and-low-risk groups. (**C**) Comparison of proportions of immune-related functions between high-and-low-risk groups. (**D**) Correlations between eight hub genes and 22 immune-related cells. (**E**) Correlations between the eight genes and immune score. (**F**) The correlation analysis between eight hub genes and 75 immune-associated genes. (**G**) Correlations between the eight genes and immune score, stromal score, estimate score. (^*^P < 0.05; ^**^P < 0.01; ^***^P < 0.001).

Figure 7A showcased an elevation in the infiltration of immune and stromal cells among patients belonging to the high-risk group in comparison to the low-risk group. To estimate the proportions of immune cells in both groups, the CIBERSORT algorithm was employed. The outcomes revealed higher proportions of Macrophages (M1), Macrophages (M2), and Eosinophils in the high-risk group, while naive B cells and monocytes were more enriched in the low-risk group (Figure 7B). Figure 7C presented the distinctions in immune-related functions between the high- and low-risk groups. The correlation between risk genes and immunity was explored, unveiling a negative association between protective genes (EXPH5, BARHL2, MAGEC3, and SLC4A9) and several immune infiltration cells, while risk genes (F2RL2, KCNMA1, B3GNT8, and CHST15) exhibited a positive association with these cells (Figure 7D, 7E). Moreover, the risk genes, including F2RL2, KCNMA1, and CHST15, displayed positive correlations with the ImmuneScore and StromalScore (Figure 7G). Lastly, Figure 7F demonstrated the reciprocal communication between the 75 immune-related genes and the eight model genes.

### Immunotherapy response prediction of risk signature

**Figure 8:**
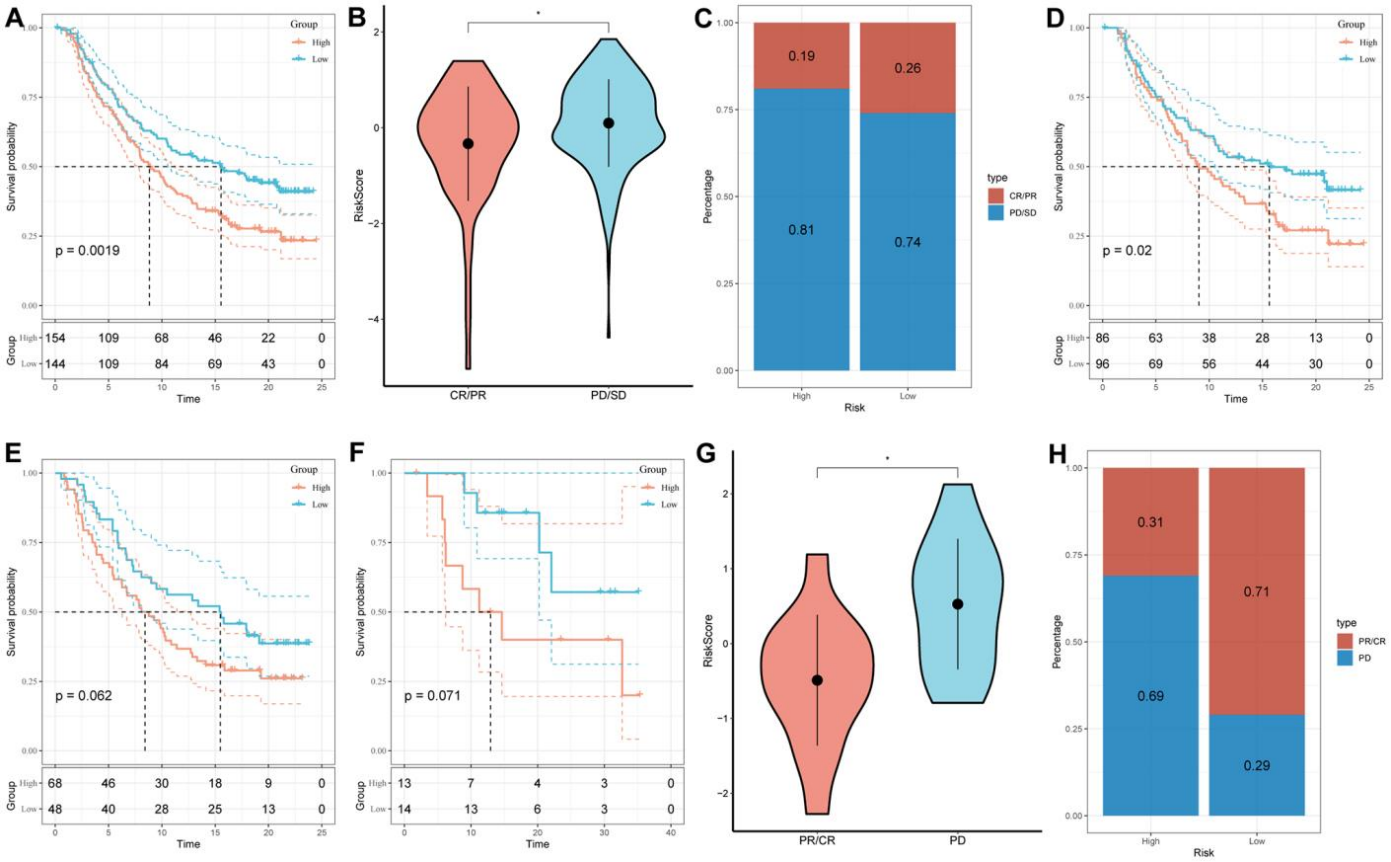
**Prediction of responsiveness to immunotherapy using our signature based on public database.** (**A**) Prognostic differences between risk subgroups in the IMvigor210 cohort. (**B**) Differences among immunotherapy responses based on risk scores in the IMvigor210 cohort. (**C**) Distribution of immunotherapy responses based on risk subgroups in the IMvigor210 cohort. (**D**) Prognostic differences between risk subgroups based on early stage (stage I–II) in the IMvigor210 cohort. (**E**) Prognostic differences between risk subgroups based on advanced patients (stage III–IV) in the IMvigor210 cohort. (**F**) Prognostic differences between risk subgroups in the GSE78220 cohort. (**G**) Differences among immunotherapy responses based on risk scores in the GSE78220 cohort. (**H**) Distribution of immunotherapy responses based on risk subgroups in the GSE78220 cohort. (^*^P < 0.05).

The assessment of the prognostic value of the immune-checkpoint therapy signature was carried out in the GSE78220 and IMvigor210 cohorts, considering the recent advancements in T-cell immunotherapy. In the IMvigor210 cohort, which included 348 patients exhibiting varying degrees of responsiveness to anti-PD-L1 receptor blockers (PD, SD, PR, and CR), Figure 8A–8C illustrated that patients in the high-risk group displayed a higher proportion of PD/SD and experienced a poorer prognosis compared to those in the low-risk group. Furthermore, SD/PD patients exhibited higher risk scores than CR/PR patients. Importantly, significant differences in survival were observed only among different risk subgroups in patients with Stage I+II disease, rather than those with Stage III+IV disease (Figure 8D, 8E). The findings were validated in the GSE78220 cohort, yielding consistent results with those of the IMvigor210 cohort; patients with PR or CR had lower risk scores and were less likely to be classified into the high-risk group (Figure 8F–8H).

### Consensus clustering and immune infiltrations analysis

**Figure 9:**
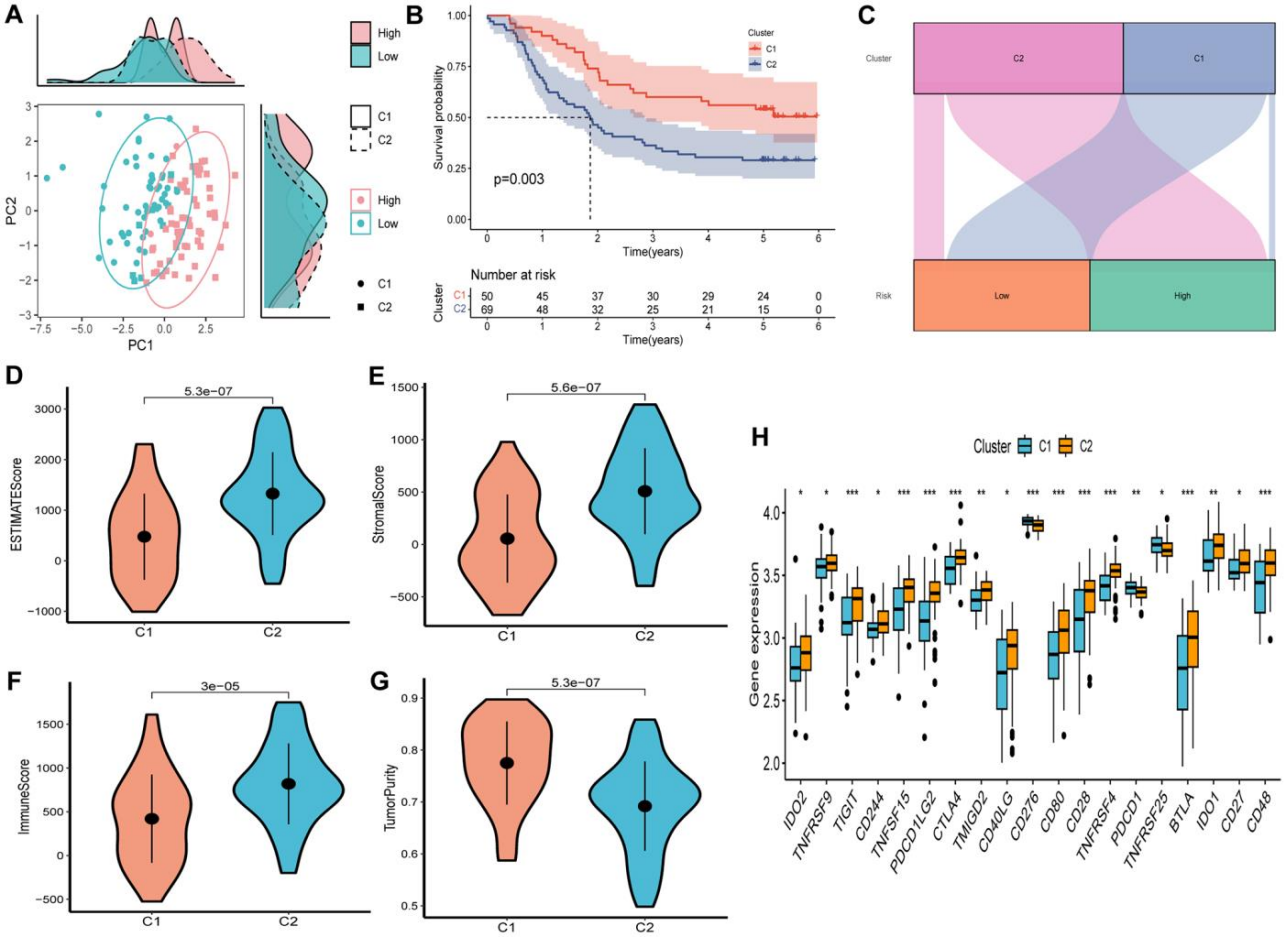
**Consensus clustering based on nine prognostic SCRGs expression.** (**A**) PCA depicted the distribution for clusters. (**B**) Survival analysis based on the two clusters. (**C**) The Sankey diagram of the connection between clusters and high-and low-risk group. (**D**) ESTIMATEScore difference between two clusters. (**E**) SromalScore difference between two clusters. (**F**) ImmuneScore difference between two clusters. (**G**) TumorPurity difference between two clusters. (**H**) Expression difference of immune checkpoints between two clusters. (^*^P < 0.05; ^**^P < 0.01; ^***^P < 0.001).

Unsupervised consensus clustering was conducted to explore the molecular subtypes based on the expression of SCRGs, which were integrated into the risk signature. By using an optimal clustering stability *k*-value of 2, the GSE53624 cohort was partitioned into two distinct clusters. The distribution of these clusters is visually presented in the ridge plot (Figure 9A). Cluster 1 (C1) exclusively comprised individuals in the low-risk group, while cluster 2 (C2) consisted of both high-risk and low-risk patients, as depicted in the Sankey diagram (Figure 9C). Subsequent survival analysis revealed that patients in the C1 group demonstrated a more favorable outcome compared to those in the C2 group (Figure 9B). TME scores were calculated for each cluster, indicating that the C2 cluster exhibited higher immune, stromal, and estimate scores, along with lower tumor purity, in contrast to the C1 cluster (Figure 9D–9G). Examination of immune checkpoint inhibitors revealed significant associations between the expression of most immune checkpoints and the C2 cluster, with the exception of CD276, TNFRSF25, and PDCD1 (Figure 9H).

### Drugs sensitivity

**Figure 10:**
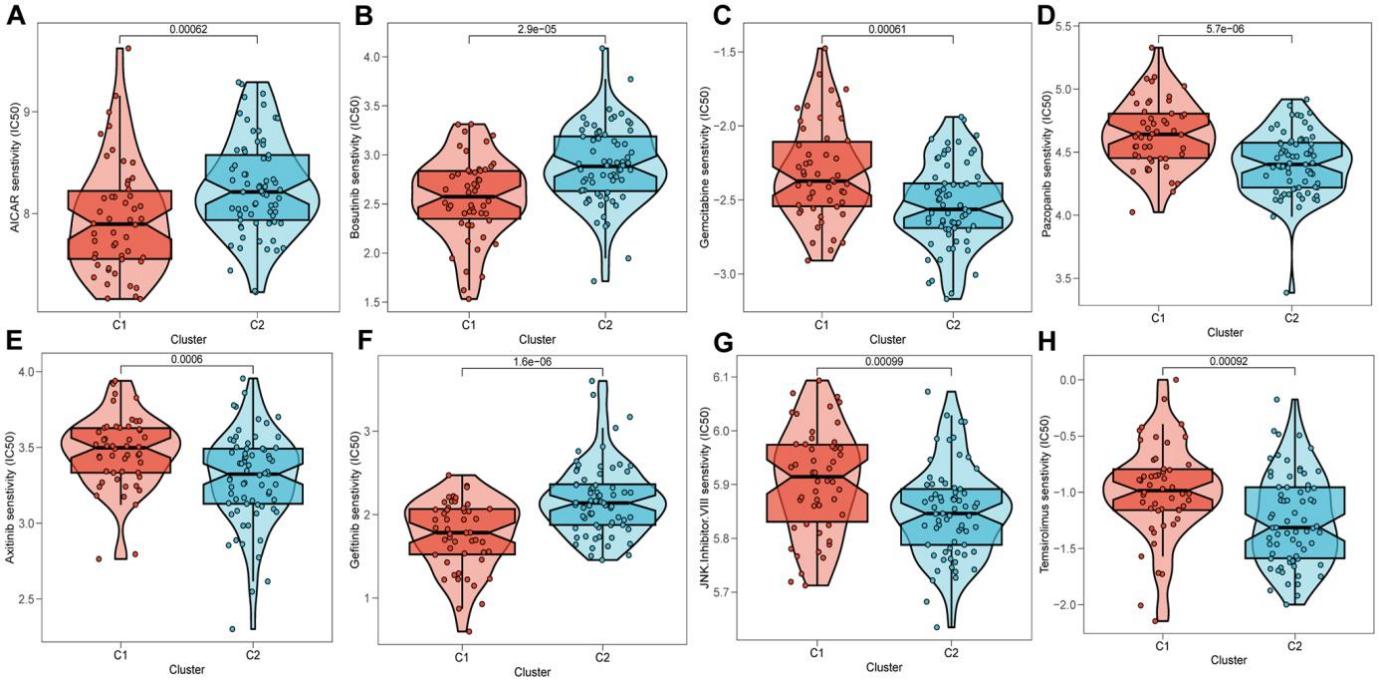
**Prediction of chemotherapy drug sensitivity in ESCC patients based on different clusters.** Chemotherapy drug sensitivity of AICAR (**A**), Bosutinib (**B**), Gemcitabine (**C**), Pazopanib (**D**), Axitinib (**E**), Gefitinib (**F**), JNK.Inhibitor.VIII (**G**), Temsirolimus (**H**).

Upon conducting an assessment of the effectiveness of various chemotherapeutic agents across distinct clusters, it has come to our attention that patients categorized under cluster 2 (C2) manifested heightened IC50 values in response to chemotherapeutic agents including Bosutinib, Gefitinib, and AICAR (Figure 10A, 10B, 10F), whereas individuals belonging to cluster 1 (C1) exhibited more favorable response rates to Gemcitabine, Pazopanib, among others (Figure 10C–10E, 10G, 10H).

### The experiment of genes involved in the risk signature

**Figure 11:**
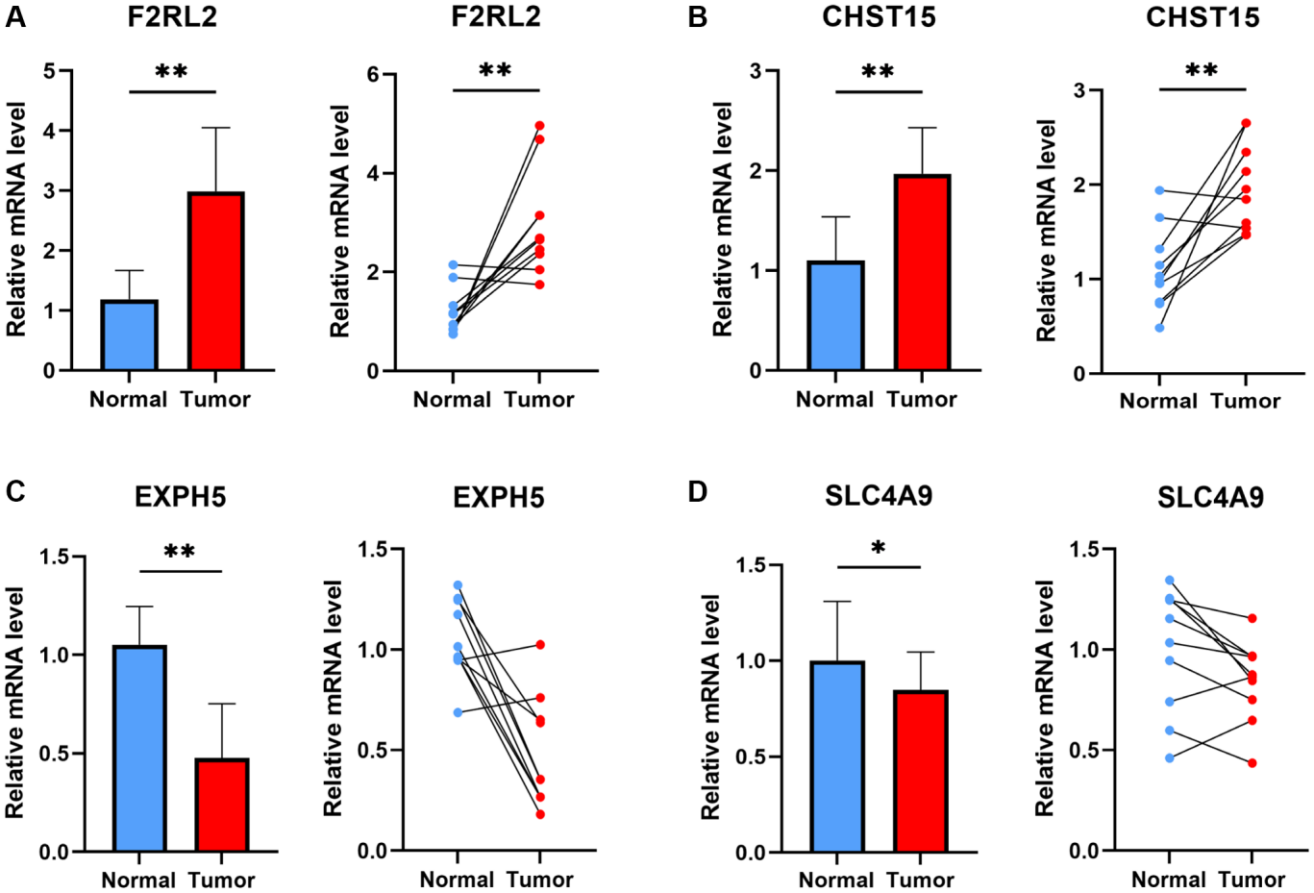
**The experiment of ESCC risk-related genes.** The expression of F2RL2 (**A**), CHST15 (**B**), EXPH5 (**C**), and SLC4A9 (**D**) in normal esophageal tissue and ESCC tissue of patients. *t*-test was used to compare the expression of genes between normal and tumor. (^*^p < 0.05, ^**^p < 0.01).

Four genes implicated in the risk signature of ESCC were selected for additional validation in ESCC patients. Figure 11 portrays the expression patterns of these genes, wherein F2RL2 and CHST15 displayed increased expression levels in tumors, while SLC4A9 and EXPH5 demonstrated significantly decreased expression levels in tumors. These observed distinctions align with our bioinformatic discoveries, suggesting the potential of these genes as novel biomarkers for early diagnosis of ESCC.

## DISCUSSION

Esophageal squamous cell carcinoma, characterized by its high malignancy, represents a substantial menace to human health as a result of limited efficacious therapeutic interventions [23]. Until September 14, 2020, the worldwide ramifications of the SARS-CoV-2 virus, known for inducing severe acute respiratory syndrome, have impacted a population surpassing 29 million, resulting in more than 900,000 fatalities. Notably, significant focus has been placed on the human cell receptor ACE2 due to its involvement in SARS-CoV-2 infection. Moreover, several studies have explored the association between ACE2 and cancer [24]. Furthermore, recent inquiries have revealed an association between the upregulation of ACE2 and improved survival outcomes in various types of cancer, including esophageal carcinoma. Moreover, apart from ACE2, the transmembrane protein AXL has been recognized for its involvement in several vital biological processes, including cell growth, migration, aggregation, metastasis, and adhesion. Importantly, this protein holds significant relevance in both the pathogenesis of COVID-19 and the advancement of cancer [25]. Moreover, recent studies have unveiled a correlation between elevated ACE2 expression and enhanced overall survival in diverse malignancies, including esophageal carcinoma. Furthermore, apart from ACE2, the transmembrane protein AXL has been implicated in various essential biological phenomena, including the facilitation of cellular growth, migration, aggregation, metastasis, and adhesion. It is worth noting that this protein plays a crucial role in both the pathogenesis of COVID-19 and the advancement of cancer [26]. Besides, one research has revealed that Diminished AGTR1 expression was frequently observed within tumors in contrast to their normal tissue counterparts, whereas AGTR2 and MAS1 exhibited notably subdued expression levels in both tissues and cell lines. The distinctive expression profiles of ACE within ovarian serous cystadenocarcinoma (OV) and kidney renal clear cell carcinoma (KIRC) demonstrated a discernible association with ubiquitin modification, facilitated by E3 ligases. Genomic alterations within the RAS gene family were comparatively rare across the TCGA pan-cancer program, with ACE displaying the highest frequency of alterations among its counterparts. The diminished AGTR1 expression might be attributed to promoter hypermethylation [27]. Hence, the association between SARS-CoV-2 and the intricate mechanisms underlying the emergence, advancement, and immunological milieu of esophageal cancer has sparked our keenness to engage in pertinent investigations.

Our investigation aims to explore the potential correlations between SARS-CoV-2 and esophageal squamous cell carcinoma by acquiring two genes that display specific expression in esophageal tissue and have associations with SARS-CoV-2, as sourced from the Human Protein Atlas. In order to identify an extensive repertoire of genes related to SARS-CoV-2, single-cell RNA-sequencing analysis was employed. Subsequently, a meticulous selection process involving differential analysis, univariate Cox regression, lasso regression, and multivariate Cox regression led to the identification of a set of eight genes (KCNMA1, F2RL2, CHST15, B3GNT8, BARHL2, EXPH5, SLC4A9, and MAGEC3). These genes were employed to construct a novel risk signature, serving as the foundation for our investigation.

Evidence suggests that KCNMA1-AS1 is significantly upregulated in epithelial ovarian cancer (EOC) tissues when compared to normal tissues. This upregulation contributes to the inhibition of apoptosis in EOC cells and acts as a prognostic indicator for adverse outcomes in patients with EOC [28], suggesting the diagnosis and prognosis prediction value that KCNMA1 has on EOC. Besides, the upregulation of KCNMA1 has been observed to promote the reversal effect of verapamil on the chemoresistance to cisplatin in esophageal squamous cell carcinoma cells [29]. The proliferative and metastatic capacities of cells in clear cell renal cell carcinoma are facilitated by CHST15 through the signaling pathway involving miR-125a-5p/EIF4EBP1, indicating its potential as a promising prognostic biomarker [30]. Like KCNMA1 and CHST15, F2RL2 and B3GNT8 have also been demonstrated to stimulate the onset and advancement of multiple malignancies [31–33]. In contrast, SLC4A9 has been identified to cause acidification in neoplastic cells and inhibit tumor progression by blocking the hypoxia-induced transport of bicarbonate, thereby exhibiting a protective role [34], which aligns with our bioinformatic discoveries. Subsequently, the GSEA analysis was employed, revealing a notable enrichment of defensive genes implicated in olfactory transduction, whereas genes linked to risk exhibited significant associations with diverse pathways, including vascular smooth muscle contraction and pathways involved in the development of cancer.

Moreover, the inquiry has yielded substantiating evidence regarding the prognostic importance of the innovative signature in individuals afflicted with esophageal squamous cell carcinoma (ESCC), as confirmed by external validation utilizing the TCGA ESCC cohort. Upon stratifying patients into high- and low-risk categories based on the median risk score, subsequent analysis has unveiled significantly superior survival outcomes in the low-risk group compared to the high-risk group. Both univariate and multivariate Cox regression analyses have established the risk score as an autonomous prognostic determinant for overall survival. Furthermore, a nomogram has been formulated employing the risk signature, exhibiting a remarkable level of concordance between predicted and observed outcomes for the overall survival of ESCC patients. These findings affirm the reliability of the risk signature as a precise prognostic tool for ESCC patients.

The remarkable progress in the field of cancer immunotherapy has provided a new outlook on cancer management, relying on a deep understanding of the immune milieu existing within the tumor microenvironment [35]. Lately, immunotherapy has effectively found application within clinical settings as an innovative modality for addressing solid tumors, instilling renewed optimism among individuals afflicted by cancer. Various immunotherapeutic approaches, exemplified by immune checkpoint inhibitors (ICIs), chimeric antigen receptor T-cell therapy, and tumor vaccines, have attained noteworthy milestones in the realm of esophageal cancer (EC) treatment. Nevertheless, the overall response rate (ORR) of immunotherapy in EC patients registers at below 30%, and a substantial proportion of patients who undergo initial immunotherapeutic intervention are predisposed to the eventual emergence of acquired resistance (AR) over the course of time. Immunosuppressive influences substantially undermine both the endurance and efficacy of immunotherapeutic modalities [36]. The significance of the biomolecule Dipeptidyl peptidase 4/CD26 (DPP4/CD26) has been proposed in elucidating susceptibility to neoplastic growth and coronaviruses, as well as its participation in the immune response [37], implying that SCRGs may exert a substantial impact on cancer immunotherapy. The neoplastic microenvironment is a complex and intricate ecosystem consisting of various cellular lineages that significantly influence cancer pathophysiology and the effectiveness of medical interventions [38]. Notwithstanding the utilization of immunomodulatory interventions, a considerable proportion of patients afflicted with esophageal squamous cell carcinoma (ESCC) continue to encounter an adverse clinical prognosis, possibly attributed to mechanisms of immune evasion or immune tolerance [39], we conducted a comprehensive analysis of the immune landscape of ESCC, based on the risk signature associated with SCRGs. Our investigation has demonstrated that the subgroup of patients classified as high-risk displays an elevated degree of immune cell infiltration. Nevertheless, our analysis has identified that the high-risk cohort is predominantly distinguished by the predominance of macrophage (M2) immune infiltrating cells, which have been validated to elicit immune evasion within the realm of cancer immunotherapy, thereby resulting in an unfavorable response to immunotherapeutic interventions [40]. Therefore, our hypothesis posits that patients categorized in the low-risk classification may encounter more advantageous outcomes with regards to immunotherapy. Subsequently, to validate our proposition, an evaluation of the IMvigor210 and GSE78220 cohorts was conducted. Our analyses unveiled that the individuals assigned to the low-risk subgroup demonstrated a heightened occurrence of partial and complete response subsequent to immunotherapy via the administration of anti-PD-L1 receptor blockade. These findings align with our prior results, thus indicating that individuals within the low-risk group appear to derive greater advantages from immunotherapy in comparison to those within the high-risk group.

The acknowledged concept of the considerable heterogeneity of esophageal squamous cell carcinoma (ESCC) is widely recognized. A comprehensive understanding of ESCC heterogeneity has the potential to bring about substantial changes in the management of this cancer, consequently leading to improved patient outcomes. To address this, consensus clustering was employed to analyze the GSE53624 cohort using a risk signature. Cluster 2, primarily composed of the high-risk group displaying a poorer prognosis, exhibited elevated immune score, stromal score, and estimate score, along with a noteworthy correlation with various immune checkpoints including BTLA, CD48, CD27, CTLA4, CD28, IDO2, among others. This suggests that patients within Cluster 2 may experience greater advantages from immunotherapy utilizing immune checkpoint inhibitors. Furthermore, it also signifies that this risk signature holds the potential to anticipate the immunotherapeutic response in a manner that extends beyond the constraints of anti-PD-1 or anti-PD-L1 treatments. Lastly, F2RL2 and CHST15 demonstrated significant overexpression in tumors, while SLC4A9 and EXPH5 exhibited significant downregulation in tumors, thereby confirming the involvement of several SCRGs indicated in the risk signature.

Despite the valuable insights gleaned from our study, there exist certain limitations that warrant careful consideration. Firstly, it is imperative to acknowledge that the development of our risk signature relied on retrospective data derived from publicly accessible databases. Thus, to mitigate potential biases, the inclusion of additional prospective and multicenter cohorts of esophageal squamous cell carcinoma (ESCC) becomes crucial. Secondly, it is vital to recognize that our risk signature specifically pertains to the prediction of response to anti-PD-L1 immunotherapy. Consequently, further investigations are warranted to evaluate its efficacy in predicting response to other targeted therapies in future applications. Lastly, to validate the functional significance of the genes implicated in the risk signature, *in vitro* and *in vivo* experiments are indispensable.

## CONCLUSION

In this study, we undertook a comprehensive investigation into the small non-coding RNA genes (SCRGs) in esophageal squamous cell carcinoma (ESCC). Through our analysis, we successfully identified eight distinct clusters of SCRGs. Notably, three of these clusters exhibited significant associations with the prognosis of ESCC. Utilizing these findings, we developed a prognostic risk signature comprising eight genes derived from the SCRGs. Furthermore, we created a novel nomogram that integrated the risk signature with clinicopathological characteristics. Impressively, this nomogram demonstrated remarkable performance in prognosticating clinical outcomes for ESCC patients. Additionally, we established a correlation between the risk signature and the immune landscape, suggesting its potential as a predictive tool for assessing the responsiveness of ESCC patients to immunotherapy targeting PD-L1 blockade. By amalgamating SCRGs with Esophageal Squamous Cell Carcinoma (ESCC), our study furnishes a fresh perspective on ESCC. Through the formulation of a risk signature, the approach to addressing this exceptionally virulent neoplasm attains a heightened level of individualization and precision.

## Supplementary Materials

Supplementary Figures
