# Supplementary material for: A SARS-CoV-2 related signature that explores the tumor microenvironment and predicts immunotherapy response in esophageal squamous cell cancer
Source: Aging (Albany NY). 2023 Oct 6;15(19):10501–23. doi: 10.18632/aging.205090 (PMC10599722; doi:10.18632/aging.205090)
Supplement: Supplementary Figures [file aging-15-205090-s001.pdf]

SUPPLEMENTARY FIGURES

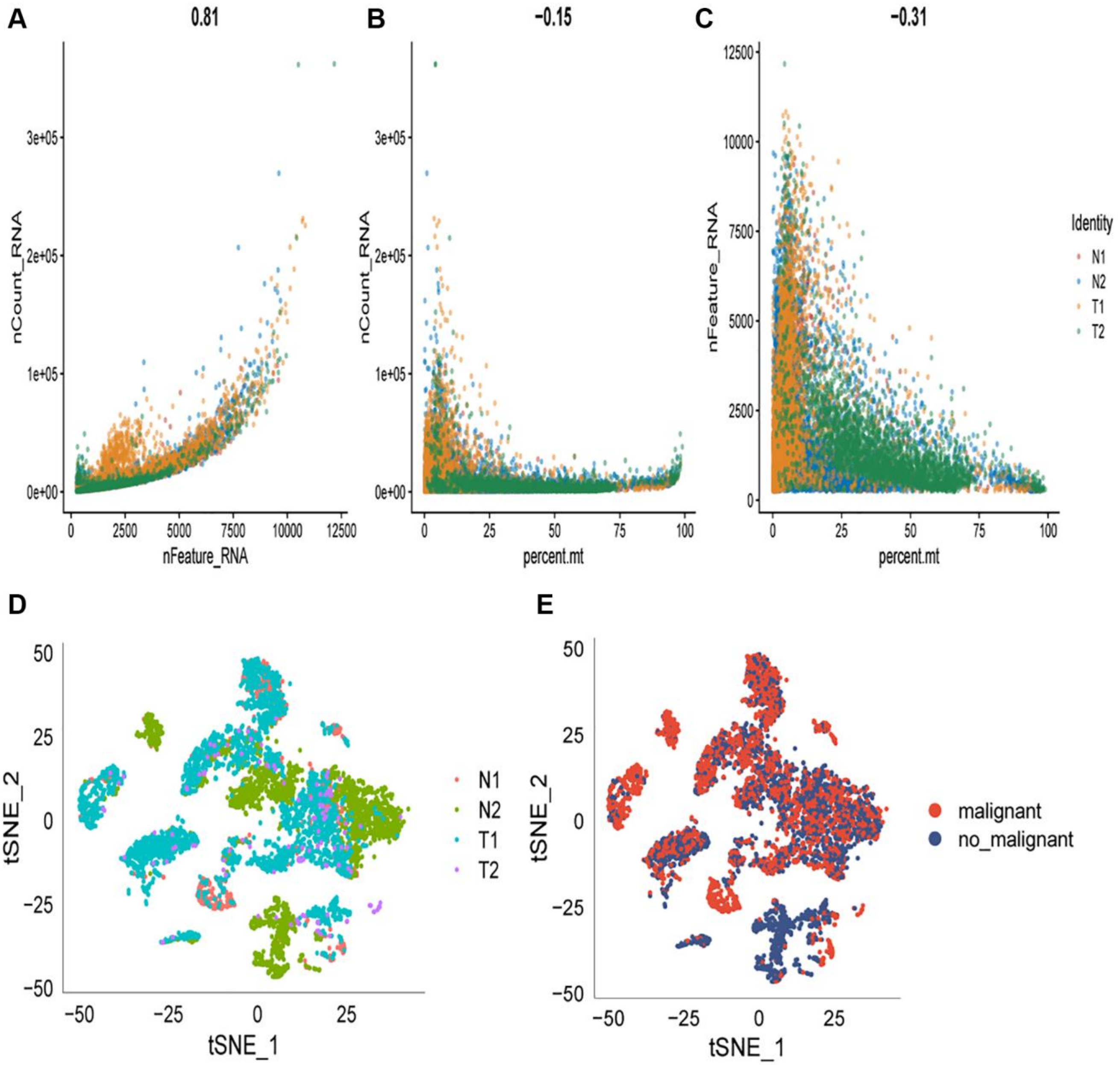

Supplementary Figure 1. The details of re-process of scRNA-seq data of ESCC. (**A**–**C**) The relationship between the amount of mRNA/UMI and mitochondrial genes, the relationship between the amount of mRNA and UMI. (**D**) tSNE distribution of different samples. (**E**) tSNE distribution of malignant and non-malignant samples.

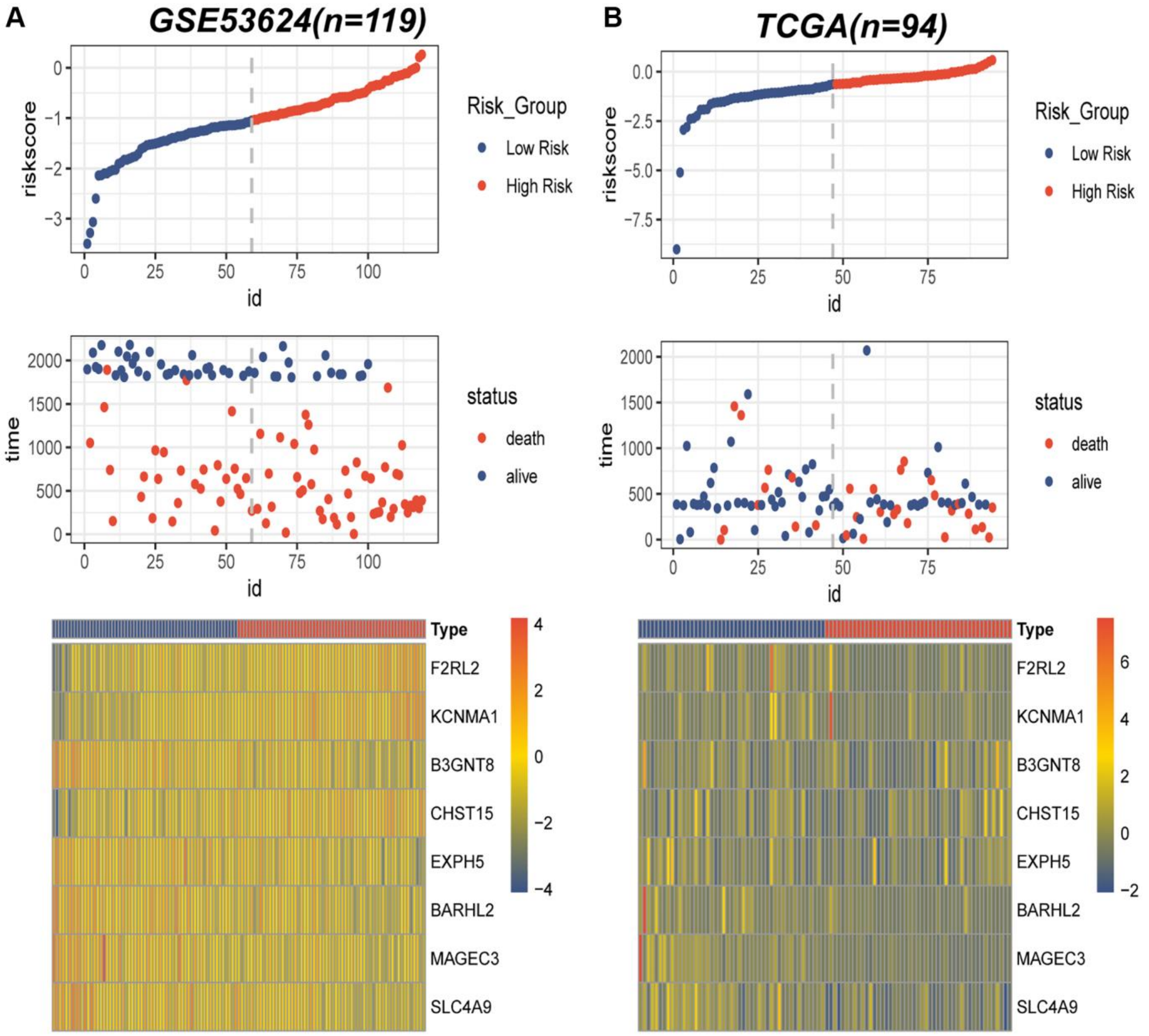

Supplementary Figure 2. Correlations between risk score and prognosis of ESCC patients. (**A**) Patient risk score distribution, scatter diagram of patient survival status, and expression pattern of prognostic genes in training cohort (GSE53624). (**B**) Patient risk score distribution, scatter diagram of patient survival status, and expression pattern of prognostic genes in test cohort (TCGA).

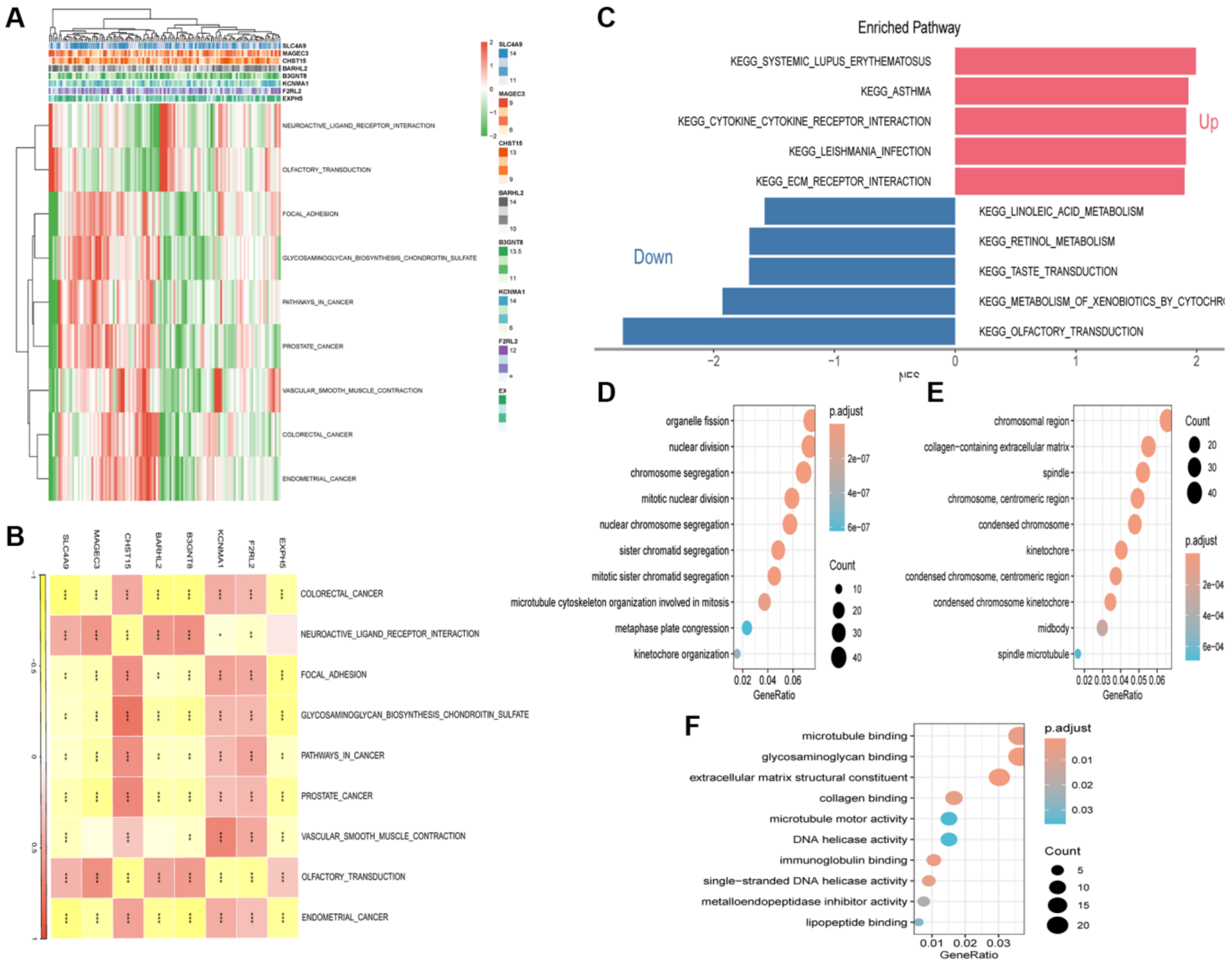

Supplementary Figure 3. Gene Set Enrichment Analysis (GSEA). (**A**) Heatmap exhibiting enrichment score for key pathways based on the hub genes. (**B**) Gene-pathway correlation heatmap. (**C**) Gene Set Enrichment Analysis of up-regulated and down-regulated genes (**D**) GO-BP analysis (**E**) GO-CC analysis (**F**) GO-MF analysis.
